# miR-2682-3p antagonizes its host lncRNA-MIR137HG by interacting with the same target FUS to regulate the progression of gastric cancer

**DOI:** 10.1186/s12885-022-09740-9

**Published:** 2022-06-22

**Authors:** Yantao Du, Yichen Chen, Tao Wu, Xiaodan Fan, Wei Lin, Zhouhua Jiang

**Affiliations:** 1 The Affiliated Hospital of Medical School of Ningbo University, Renmin Road No.247, Ningbo, 315020 Zhejiang China; 2 Ningbo Institute of Medical Science, Yangshan Road No.42-46, Ningbo, 315020 Zhejiang China; 3 Medical School of Ningbo University, Fenghua Road No.818, Ningbo, 315211 Zhejiang China; 4 Zhejiang Pharmaceutical College, Ningbo, 315100 ZhejiangZhejiang China; 5 Ningbo Medical Centre Lihui Li Eastern Hospital, Ningbo University, Jiangnan Road No.1111, Ningbo, 330212 Zhejiang China; 6 Ningbo Women and Children Hospital, Ningbo Liuting Road No.339, Ningbo, 315012 Zhejiang China

**Keywords:** LncRNA, MIR137HG, miR-2682-3p, FUS, Gastric cancer

## Abstract

**Background:**

The mechanism of long non-coding RNA MIR137HG in human gastric cancer (GC) is currently unknown. In the present study, we aimed to explore the function and mechanism of MIR137HG in gastric cancer.

**Methods:**

The expression of lncRNA-MIR137HG in 69 gastric cancer samples and their paired surgical margin (SM) tissue samples were tested by QRT-PCR. UCSC was used to find the gene location relationship among MIR137HG and its embedded miRNAs. TargetScan was used to predict the targets of miR-2682-3p. Starbase was used to predict the candidate proteins that interacted with MIR137HG. Western blot, co-focus, and RIP assay were used to verify the direct interaction between MIR137HG and FUS (fused in sarcoma/translocated in liposarcoma, FUS/TLS), while dual-luciferase reporter assay was used to confirm the interaction between miR-2682-3p and FUS. Cell migration assays, colony formation, and xenografts assay were used to investigate the function of MIR137HG and miR-2682-3p to tumor growth and metastasis. Western blot assay was used to explore the downstream candidate protein of FUS.

**Results:**

Data showed that MIR137HG expressed significantly higher in GC than in SM. MIR137HG promoted colony formation and migration in vitro and promoted tumor formation and metastasis in vivo. MIR137HG is distributed in both the nucleus and cytoplasm. It was co-located with FUS and could directly interact with FUS, which might interact with other proteins, such as MET(MET-proto-oncogene, receptor tyrosine kinase), RHOC(ras homolog family member), and CTNNB1(catenin beta1). These proteins may involve different signaling pathways to regulate gastric cancer progression. By contrast, the embedded miR-2682-3p could antagonize the series functions of its host lncRNA-MIR137HG by targeting FUS.

**Conclusions:**

lncRNA-MIR137HG promoted growth and metastasis in gastric cancer by interacting with FUS, while miR-2682-3p could inhibit the function of MIR137HG via the same target FUS.

**Supplementary Information:**

The online version contains supplementary material available at 10.1186/s12885-022-09740-9.

## Background

Gastric cancer (GC) is a high-recurrence-rate malignancy carcinoma, the third major cause of cancer-related death worldwide, second only to lung and liver cancer [1, 2]. Data showed that the incidence and mortality of GC in Asian countries are increasing year after year, especially in the countries of East Asia, such as China, Japan, and Korea [3, 4]. In the last few decades, significant progress has been made in GC treatment, and many attempts have been made to find effective treatment strategies. However, the morbidity and mortality of GC remain at a high level. Therefore, gaining insight into the underlying mechanisms of GC progression will help find the novel diagnosis and treatment for GC.

Non-coding RNAs play essential roles in keeping cells in their state, and their abnormal expression might lead to various diseases, including cancer. Among them, long non-coding RNAs (lncRNAs), which are more than 200 nucleotides in length, have been identified different vital functions, such as participating in the autophagy pathway [5, 6], controlling cell differentiation [7], and acting as competing endogenous RNAs (ceRNAs) of miRNAs [5, 8]. In addition, sometimes lncRNA can be the host gene of miRNAs [9] which makes a more interesting relationship between them. For example, MIR100HG, the host gene of miR-125b, was inhibited by GATA6, but this repression will be relieved by miR-125b by targeting GATA6 [10].

MIR137HG, the host gene of miR-137 and miR-2682, is a long non-coding RNA. It was primarily reported in neural system diseases. For example, it was reported that polymorphism of MIR137HG rs1625579 was related to schizophrenia [11, 12]. Besides, researchers have found that MIR137HG could also influence human cancer. According to the TCGA database analysis, Liu and colleagues found that MIR137HG could play as ceRNA in Laryngeal squamous cell carcinoma (LSCC) [13] and muscle-invasive bladder cancer (MIBC) [14]. However, to date, the function of MIR137HG and its relationship with embedded miRNAs in gastric cancer has not been investigated.

miRNA is another kind of small non-coding RNA. They can regulate the coding gene expression at the transcriptional level and interact with long non-coding RNAs. The gene of miR-2682 is located in the second intron of MIR137HG, which has been reported in some cancers. For example, it has been reported that miR-2682-3p could inhibit osteosarcoma cell proliferation by targeting CCND2 (Cyclin D2), MMP8 (Matrix metallopeptidase 8), and Myd88 (Myeloid differentiation primary response gene 88) [15]. miR-2682-5p can inhibit HOXB8 (Homeobox B8) from promoting the function of LNC01006 [16]. miR-2682-5p could also form a feedback cycle with ETS transcription factor ELK1 (ELK1)/lncRNA-SNHG7 to enhance bladder cancer cell growth [17]. However, the function of miR-2682 has not been reported in gastric cancer. Therefore, it became the second target in our present study.

Fused in Sarcoma/Translocated in Sarcoma (FUS) is a critical nucleus RNA-binding protein. It can shuttle back and forth between the nucleus and cytoplasm. It has been reported as an essential gene in neural system diseases, such as Amyotrophic lateral sclerosis (ALS). For example, cytoplasmic FUS aggregates are a pathological hallmark in a subset of patients with ALS [18]. FUS has also been reported in cancer. circ0005276 interacted with FUS to activate the transcription of its host gene XIAP (X-linked inhibitor of apoptosis protein) to regulate the process of prostate cancer [19]. Dian Xiong and colleagues indicated that elevated FUS/TLS expression is negatively associated with E-cadherin expression and prognosis of patients with non-small cell lung cancer [20]. Qiong Wu et al. reported that DLX6-AS1 promotes cell proliferation, migration, and EMT of gastric cancer through FUS-regulated MAP4K1 [21]. Hong Zhu et al. have reported that XIST(X inactive specific transcript) served as a ceRNA in cervical cancer progression through modulating miR-200a/Fus axis [22]. However, FUS plays as the same target by both lncRNA and its embed miRNA have not been reported. Therefore, it became the third target in our present study.

The present study found that MIR137HG was overexpressed in gastric cancer (GC) samples than surgical margin (SM) samples. It could promote the abilities of migration, invasion, and colony formation in gastric cancer cell lines. At the same time, the expression of miR-2682-3p was negatively related to the expression of MIR137HG and could inhibit the migration and colony formation in gastric cell line caused by MIR137HG. The animal model confirmed the negative relationship between MIR137HG and miR-2682-3p. FUS was the exact target of both MIR137HG and miR-2682-3p, which can interact with series proteins, including CTNNB1, RHOC, MET. In conclusion, MIR137HG, miR-2682-3p, and FUS formed a negative feedback loop to regulate gastric cancer progression.

## Methods

### Sample collection and cell lines culture

Sixty-nine GC samples and their paired surgical margin samples were collected from the Affiliated Lihui Li Hospital of Ningbo University between 2016-2017. Samples were stored at liquid nitrogen (-196℃). All individuals provided informed written consents for using their tissues in this experimental study, and the Ethics Committee of Ningbo University approved this study.

The cell line BGC823 was obtained from Beijing Cancer Hospital. HGC27 was obtained from the Medical School of Ningbo University. Both BGC823 and HGC27 were tested by STR authentication (GENEWIZ, China).

### Quantitative real-time PCR

According to the manufacturer’s protocol, the total RNA of tissue samples and gastric cancer cell lines was extracted by Trizol reagent (Life Technologies, Carlsbad, USA). cDNA was synthesized by HiFiScript cDNA synthesis Kit (CW2569, CWBIO. China). RNA polymerase (M0276S, NEB, USA) and RT-adaptor were needed for miRNA-specific reverse transcription. The sequences of primers were as follows: RT-adaptor, CGAGCACAGAATTAATACGACTCACTATAGGTTTTTTTTTTTTVN; miR-2682-3p PF, CGCCTCTTCAGCGCTGTCTTCC; U6 PF, CGCTTCGGCAGCACATATAC; U6 PR, TTCACGAATTTGCGTGTCAT; MIR137HG PF, CAAGGCATCCAAAGCCTCT; MIR137HG PR, TGTGGTGAGTCAAGATCACGTC; GAPDH PF, GAAGGTGAAGGTCGGAGT; GAPDH PR, GAAGATGGTGATGGGATTTC; Universal miRNA PR, GCGAGCACAGAATTAATACGAC; miR-137 was amplified by All-in-One miRNA qRT-PCR Detection kit according to the manufacturer’s protocol (QP015, GeneCopoeia, USA), using corresponding primer U6 (HmiRQP9001, GeneCopoeia, USA), miR-137 (HmiRQP0175, GeneCopoeia, USA).

### Construction of control lentivirus and MIR137HG lentivirus cell strain in GC cell lines

MIR137HG was synthesized and cloned onto pcDNA 3.1b vector by Sangon Biotech Inc. It was then packaged as MIR137HG lentivirus and negative control lentivirus by HANBI Inc. Lentivirus was infected into BGC823 and HGC27 cell lines for 48 h and set up stable strains by puromycin (0.25 μg/ml).

### Western blotting

Total proteins were extracted from a cell by RIPA lysis buffer, and the BCA procedure determined protein concentration (C503021, Sangon Biotech. China). The protein (20 μg) was electrophoresed by 12% SDS-PAGE and transferred onto PVDF membranes. The membranes were blocked for 2 h at room temperature with 5% non-fat milk in PBST. The membranes were then incubated overnight at 4℃ with rabbit anti-TLS/FUS (ab23439,1:1000, Abcam, Cambridge, UK), rabbit anti-MET(8198, 1:1000, CST, USA), rabbit anti-RHOC (3430, 1:500, CST, USA), rabbit anti-CTNNB1(8480,1:500, CST, USA), Mouse anti-GAPDH (ab8245, 1:1000, Abcam, Cambridge, UK) and rabbit anti-ACTB (AC026, 1:500, ABclonal, China), followed by incubated with HRP-conjugated Goat Anti-Rabbit IgG (D110058, 1:2000, BBI, Sangon Biotech, Shanghai, China) or HRP-conjugated Goat Anti-Mouse IgG (D110087, 1:2000, BBI, Sangon Biotech, Shanghai, China) at room temperature for 1 h. The targeted proteins were then analyzed by LI-COR model 3600(LI-COR, USA).

### Cell migration assays (transwell assay and scratch assay)

About 4 × 10^4^ cells were seeded within 200 μl RPMI1640 onto the upper layer of the chamber. The lower layers were added about 500 μl mixed cultures with 90% RPMI 1640 and 10% FBS. 24 h later, cells were fixed by 2% formaldehyde for 20 min, followed by washing twice by 1 × PBS, and then stained with 1% crystal for 30 min. Cells in the upper well were removed with cotton swabs, and cells on the opposite membranes of the chamber were counted by the microscope system. Each chamber was counted in six fields. BD Matrigel (356234, USA) was diluted by RPMI1640 (1:8) and was seed 80 μl per hole for the invasion assay.

Cells were plated to 70% confluence on 6 well plates and were wounded with 200ul pipette tips. The wound healing status of each group was observed and photographed after scratching 0 and 18 h. 

### Dual-Luciferase reporter assay

The 3’-UTR segments of the WT and MUT FUS gene were amplified by polymerase chain reaction (PCR) and inserted into PmiR-PB-ReportTM. Co-transfections of WT-FUS-3’-UTR or MUT-FUS-3’-UTR plasmids with miR-2682-3p mimic into the cells were accomplished by using Lipo6000TM. Luciferase activity was measured after 48 h transfection by the Dual-Luciferase Reporter Assay System (E2920, Promega, USA). All assays were performed in triplicated, and each experiment was repeated three times.

### Animal models

The experiments were reviewed and approved by the Laboratory Animal Ethical Committee at Ningbo University. Five-week-old female Balb/C nude mice (*n* = 18) were purchased from SLAC.cn (Shanghai, China), and maintained in a specific pathogen-free environment. For tumor formation assay, 1 × 10^7^ GC cells were subcutaneously implanted in nude mice. After 15 days, mice were sacrificed, and tumors were removed for examination. We first injected 1 × 10^7^ GC cells for 7 days for the tumor inhibition assay to form a subcutaneous MIR137HG tumor model and injected the Ago-miR-2682-3p (1 nmol/ time) every 3 days into the tumor model and tested the volume. After 14 days, mice were sacrificed, and tumors were removed for examination. For the metastasis assay, 1 × 10^7^ GC cells were injected into the tails caudal of nude mice. After 42 days, mice were sacrificed, and their lungs were removed for examination.

### Immunohistochemistry (IHC)

Paraffin-embedded tissue sections were deparaffinized and rehydrated for IHC with the following procedures: tissue glass slides were immersed in Citrate Buffer (PH 6.0) to repair the antigen. Slides then were successively blocked by 3% hydrogen peroxide and 3% BSA, incubated with primary antibodies overnight at 4℃(FUS: Ab70381, 1:1000, Abcam, Cambridge, UK; KI67: 1:100, A20018, Abclonal, China). Subsequently, the HRP-goat anti-rabbit secondary antibody (1:200, GB23303, Sevicebio, China) was applied and incubated for 1 h at room temperature. The expression of FUS and KI67 was visualized by using DAB (K5007, DAKO, Denmark) and counterstained with hematoxylin (G1004, Servicebio, China).

### RNA-FISH and RNA/protein co-location

RNA-FISH kit (R11010.2, RICOBIO.Co., Ltd. Guang Zhou, China) was used to evaluate the sub-location of MIR137HG in cell lines, following the manufacturer’s instructions. For the co-sub-location of MIR137HG and FUS, we added 3 steps into the manufacturer’s instructions: Cells were blocked by DEPC-PBS buffer contains 5% BSA, 0.1% triton after the RNA-FISH prehybridization step for 1 h; The anti-Rabbit FUS antibody (ab70381, 1:300, Abcam, Cambridge, UK) and MIR137HG probe (lnc1100344, RICOBIO.Co., Ltd. Guang Zhou, China) were incubated together overnight at 37 ℃; The Alexa Fluor 488-conjugated Goat anti-rabbit IgG (1:500, Abcam, Ab150077) was incubated 1 h at 37℃, followed by 1×PBS washing for 3 times. The results were tested by a confocal microscope. 

### RNA immunoprecipitation

The binding of MIR137HG with FUS and DGCR8 was confirmed through the RIP kit according to its manufacturer’s protocol (Millipore/17-701/EZ-Magna RIPTM RNA-Binding Protein Immunoprecipitation Kit, Millipore Corporation, Billerica, MA01821, USA). The enrichment of MIR137HG was assessed via real-time quantitative PCR.

### IP and LC–MS/MS

About 2 × 10^7^ BGC823 MIR137HG cell strain was washed with pre-chilled phosphate-buffered saline (PBS) and lysed by RIPA (R0010-20, Solarbio, China). Cell lysates were scratched and centrifuged at 14000 g for 15 min at 4℃ to separate the supernatant. The supernatant was incubated with 100 μl protein A/G beads for 10 min at 4℃ and centrifuged 14000 g for 15 min at 4℃ to get the supernatant. The supernatant was tested the protein concentration by BCA Kit (C503021, Sangon Biotech, China). Protein was diluted by 1×PBS to 2 μg/ul, and separately incubated with rabbit anti-FUS (5 μg, Ab70381,Abcam, UK) and rabbit anti-IgG (PP64B, Millipore, USA) antibody overnight at 4℃. Subsequently, 100ul protein A/G was incubated with the complex overnight at 4℃. Collecting the complex and performed the SDS-PAGE and stained by Coomassie Blue Staining Solution (P1305-1, Solarbio, China). Protein strip around 75KD (location of FUS) was then sent to Sangon Biotech for LC-MS/MS analysis. 

### Bioinformatic analysis

The location of MIR137HG and its host miRNAs (miR-137 and miR-2682) were analyzed by UCSC (http://genome.ucsc.edu/). The prediction of candidate target proteins of MIR137HG and miR-2682-3p were separately performed using the online tools: Starbase [23, 24] (http://starbase.sysu.edu.cn/) and TargetScan (http://www.targetscan.org). In addition, the interaction among target proteins was predicted by String (https://string-db.org/).

### Statistical analysis

All statistical analyses were conducted using SPSS 18.0 statistical software. An intergroup comparison was carried out using Mann-Whitney *U*-test or the Students’ *t*-test according to data type. A receiver operating characteristics (ROC) curve was generated using the expression level of MIR137HG to separate GC patients with poor differentiation from GC patients with moderate/well differentiation. Differences with a *P* < 0.05 were considered statistically significant.

## Results

### MIR137HG levels were increased in gastric cancer

**Fig. 1:**
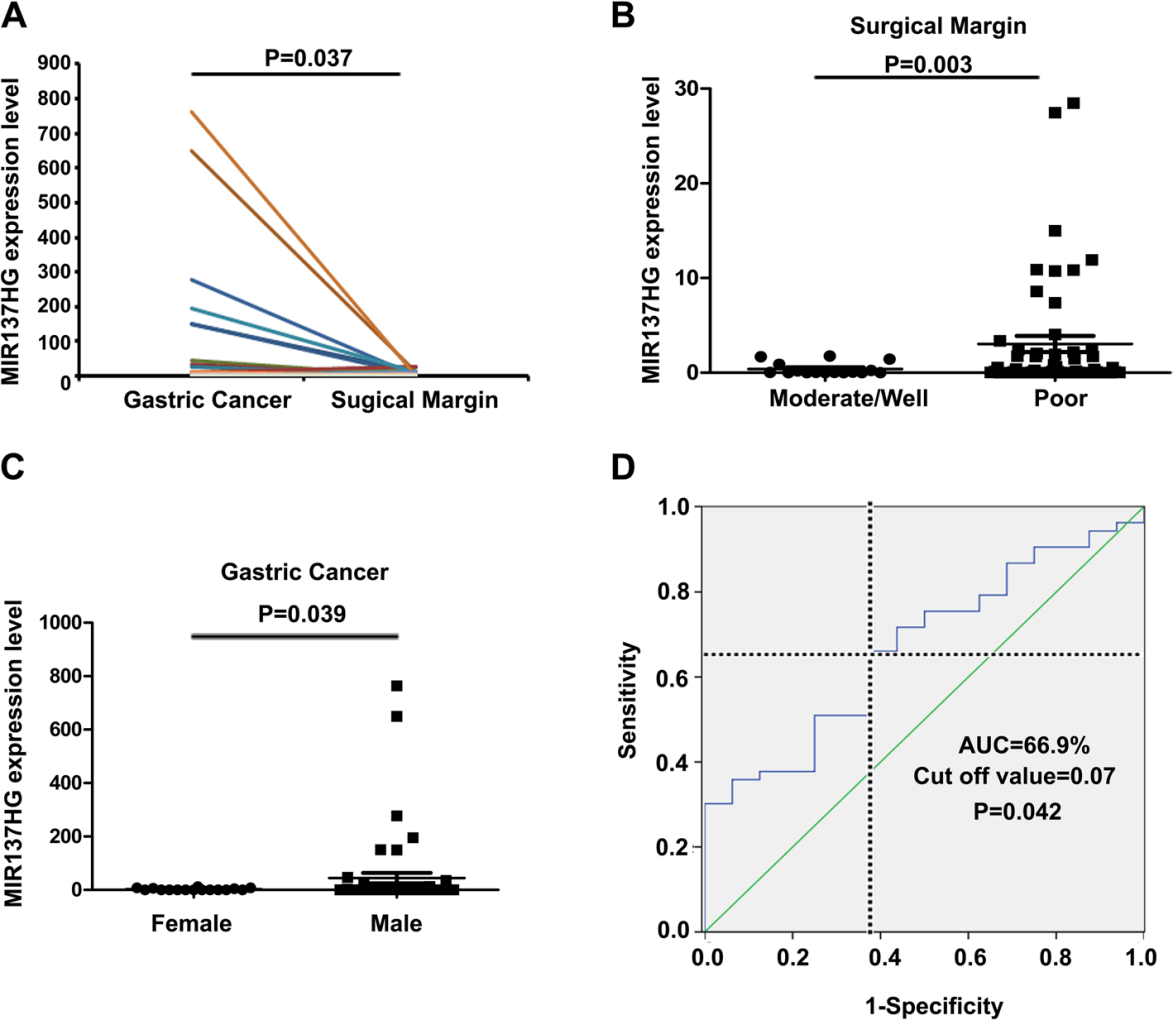
Expression analysis of MIR137HG in GC patients. **A** QRT-PCR analysis of MIR137HG expression in 69 paired gastric cancer (GC) and surgical margin (SM) samples (Paired t-test: *P* = 0.037); **B **Compration the expression of MIR137HG in SM samples between GC patients with poor differentiation and moderate/ well differentiation (Mann–Whitney *U*-test: *P* = 0.003); **C** Compration the expression of MIR137HG in GC samples between male patients and female patients (Mann–Whitney *U*-test: *P* = 0.039); **D** ROC curve used for discriminating GC patients with with poor differentiation from moderate/ well differentiation( AUC = 66.9%, Cutoff value = 0.07, *P* = 0.042)

69 gastric cancer (GC) tissue samples and their paired surgical margin (SM) tissue samples were tested by quantitative real-time polymerase chain reaction (QRT-PCR). Data showed that the expression of MIR137HG was upregulated in GC samples compared with SM samples (Fig. 1A, Student’ *t*-test, GC VS SM, *P* = 0.037). Further analysis showed that MIR137HG expressed higher in patients with low tissue differentiation than in patients with moderate/high tissue differentiation in the SM group. (Fig. 1B and Table 1, Mann–Whitney *U* -test, Poor VS Moderate/High, *P* = 0.003). MIR137HG was increased in male patients compared with female patients in the GC group (Fig. 1C and Table 1, Mann–Whitney *U*-test, Male VS Female, *P* = 0.039). The discrimination of GC patients with poor differentiation from GC patients with moderate/well differentiation by using MIR137HG expression in SM samples was demonstrated by the ROC curve with an area under the curve (AUC) of 0.667 (Fig. 1D, Cut off value = 0.07, *P* = 0.042).

**Table 1:** Clinical features of MIR137HG expression in gastric cancer samples and paired surgical margin samples

Clinical features	Classic	Case no	GC Expression Median[25%-75%]	*P*-value	SM Expression Median[25%-75%]	*P*-value
**Age (Years)**	** ≤ 60**	**23**	**0.036 [0.002–1.743]**	**0.556**	**0.162 [0.005–1.735]**	**0.603**
	** > 60**	**46**	**0.097 [0.006–6.897]**		**0.109 [0.014–1.808]**	
**Sex**	**Male**	**53**	**0.107 [0.006–2.962]**	0.039*	**0.504 [0.045–3.321]**	**0.388**
	**Female**	**16**	**0.048 [0.003–6.071]**		**0.135 [0.009–1.706]**	
**Differentiation**	**Poor**	**53**	**0.095 [0.005–5.610]**	**0.676**	**0.239 [0.024–2.318]**	0.003*
	**Moderate/ Well**	**16**	**0.024 [0.004–2.833]**		**0.034 [0.002–0.720]**	
**Vascular emblus**	**No**	**27**	**0.228 [0.018–11.426]**	**0.781**	**0.560 [0.044–1.947]**	**0.349**
	**Yes**	**42**	**0.042 [0.003–2.784]**		**0.077 [0.002–1.710]**	
**pTNM stage**	**I-II**	**30**	**0.108 [0.004–4.554]**	**0.809**	**0.142 [0.024–1.742]**	**0.290**
	**III-IV**	**39**	**0.078 [0.007–4.686]**		**0.155 [0.008–1.947]**	
**Depth of invasion**	**T**_**1+2**_	**21**	**0.163 [0.009–11.008]**	**0.385**	**0.237 [0.024–1.748]**	**0.472**
	**T**_**3+4**_	**48**	**0.066 [0.004–3.004]**		**0.141 [0.009–1.890]**	
**Lymh node metastasis**	**No**	**21**	**0.108 [0.004–6.230]**	**0.063**	**0.193 [0.026–1.749]**	**0.192**
	**Yes**	**48**	**0.082 [0.005–4.276]**		**0.151 [0.009–1.890]**	
**Distant metastasis**	**No**	**66**	**0.091 [0.005–5.148]**	**0.632**	**0.159 [0.010–1.808]**	**0.479**
	**Yes**	**3**	**NI**		**NI**	

^*^ Mann–Whitney *U* -test: *P*<0.05; *GC*:Gastric Cancer; *SM*:Surgical Margin; *NI*:No information

### MIR137HG promote cell colony formation and cell migration in gastric cancer cell lines

**Fig. 2:**
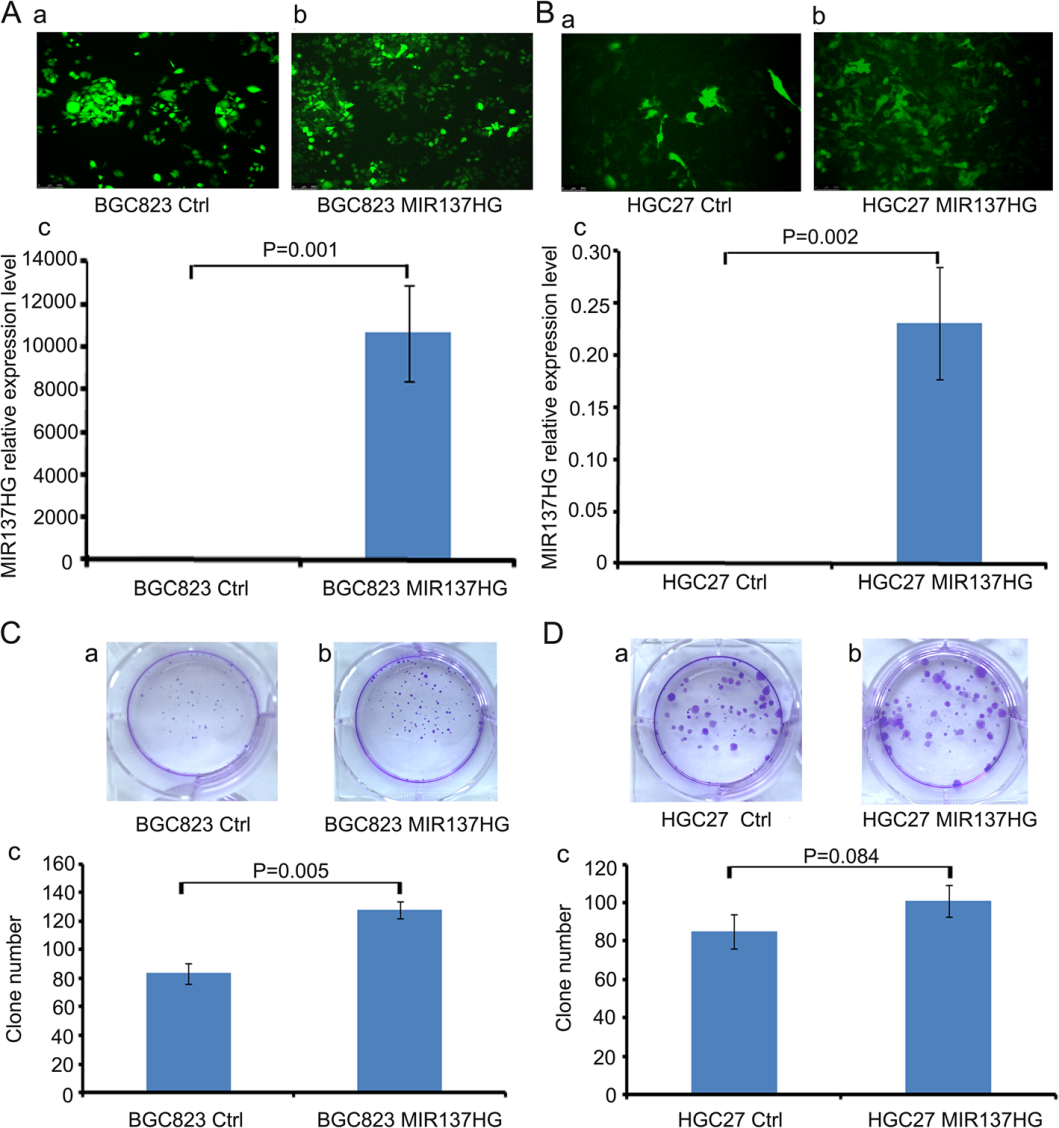
Construction of MIR137HG over-expression cell lines and the colony formation assay. **A** and **B** Construction of MIR137HG over-expression cell lines (**A** a and b BGC823 Ctrl and MIR137HG, *P* = 0.001; **B** a and b HGC27 Ctrl and MIR137HG, *P* = 0.002). **C** and **D** Colony formation assay (**C**a and b BGC823 Ctrl and MIR137HG, *P* = 0.005; **D**a and b HGC27 Ctrl and MIR137HG, *P* = 0.084)

**Fig. 3:**
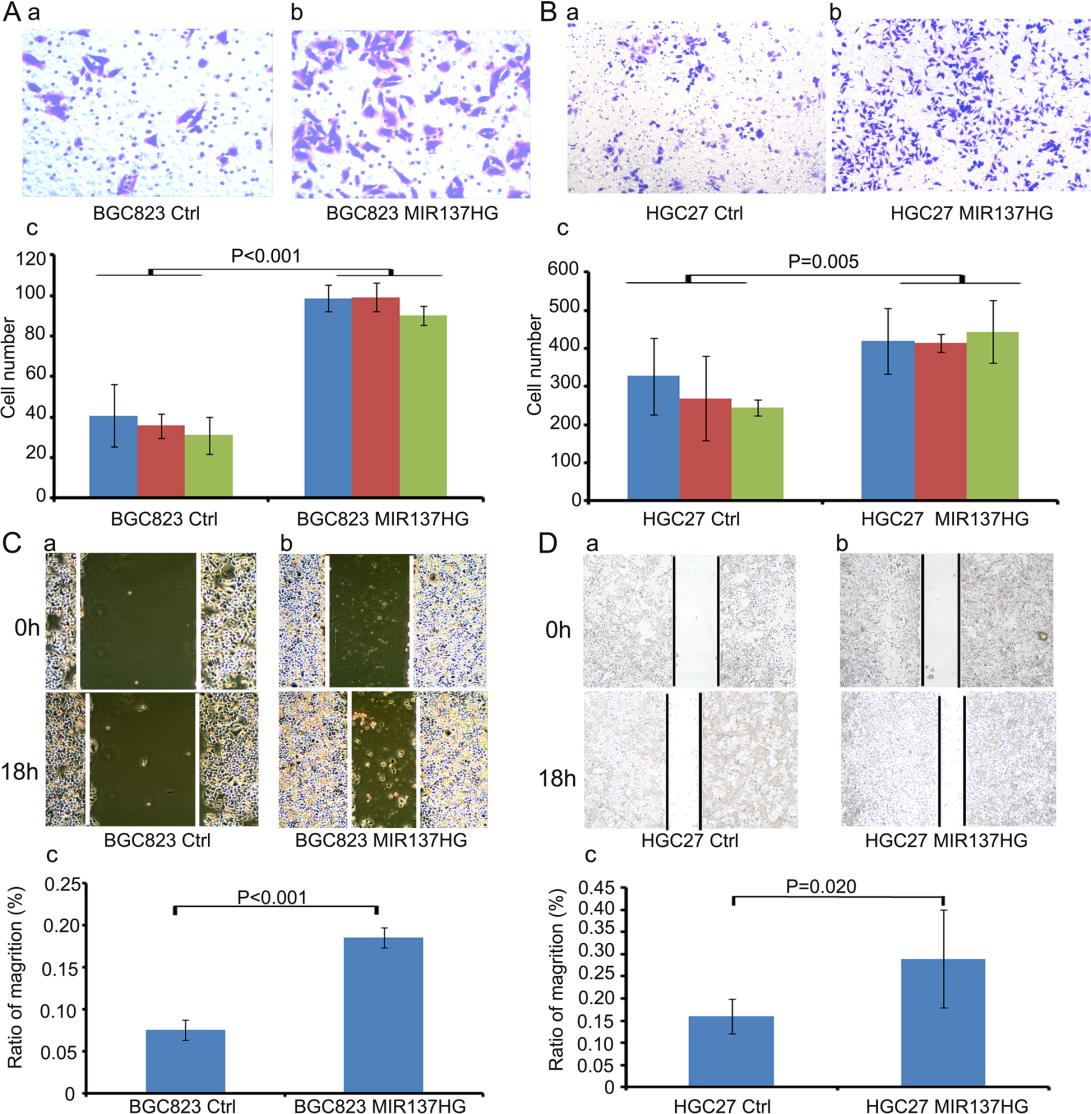
Transwell and Scratch assays. **A** and **B** Transwell assay (**A** (a,b,c): BGC823 Ctrl and MIR137HG, *P* < 0.001; **B** (a,b,c): HGC27 Ctrl and MIR137HG, *P* = 0.005). **C** and **D** Scratch assay (**C**(a,b,c): BGC823 Ctrl and MIR137HG, *P* < 0.001; **D**(a,b,c), HGC27 Ctrl and MIR137HG, *P* = 0.020)

To investigate the function of MIR137HG in gastric cancer, we infected MIR137HG over-expression lentivirus in BGC823 and HGC27 (Fig. 2A (a,b,c), *P* = 0.001 and Fig. 2B (a,b,c), *P* = 0.002). Colony formation assay showed that BGC823 MIR137HG could significantly promote colony formation compared to controls (Fig. 2C (a,b,c), *P* = 0.005). HGC27 MIR137HG could also boost the colony formation compared with its control, but not significantly (Fig. 2D (a,b,c), *P* = 0.084). Cell migration ability was separately tested by transwell assay and scratch assay. Data showed that both BGC823 MIR137HG and HGC27 MIR137HG could boost the cell migration ability compared with their controls (Fig. 3A (a,b,c) and B (a,b,c), Transwell assay, *P* < 0.001 in BGC823, *P* = 0.005 in HGC27; Fig. 3C(a,b,c) and D(a,b,c), Scratch assay, *P* < 0.001 in BGC823, *P* = 0.020 in HGC27).

### The embedded miR-2682-3p could antagonize the function of MIR137HG in vitro and in vivo

**Fig. 4:**
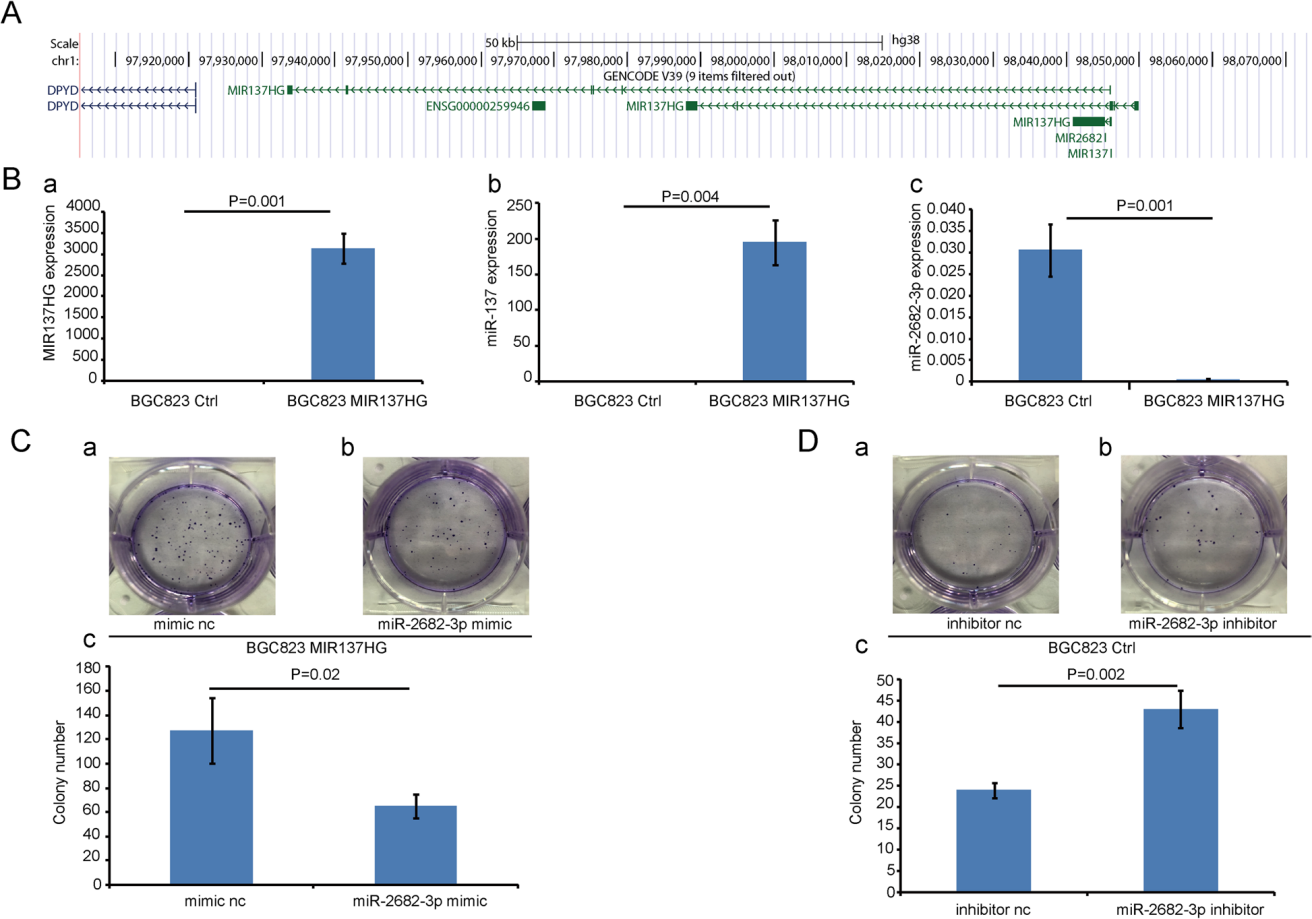
The relationship between miR-2682-3p and MIR137HG. **A** UCSC database indicated the gene location of miR-137, miR-2682, and MIR137HG. Data showed that miR-2682 was embedded at the second intron of gene MIR137HG, while miR-137 was embedded at the third exon of gene MIR137HG **B **The expression relationship among MIR137HG, miR-137, and miR-2682-3p was tested by QRT-PCR. Data showed that MIR137HG was positively related to miR-137 (*P* = 0.004), while it was negatively related to miR-2682-3p (*P* = 0.001). **C** and **D** showed the colony formation assays. Data showed that miR-2682-3p mimic could inhibit the colony formation of BGC823 MIR137HG (*P* = 0.02), while miR-2682-3p inhibitor could promote the colony formation of BGC823 Ctrl (*P* = 0.002)

According to the UCSC database, miR-137 and miR-2682 are separately located in the third exon and the second intron of the MIR137HG gene (Fig. 4A). So we were interested in their expression relationship. We found that the expression of miR-137 was positively related with MIR137HG (Fig. 4Ba, *P* = 0.001 and Fig. 4Bb, *P* = 0.004), while the expression of miR-2682-3p was negatively associated with MIR137HG expression (Fig. 4Ba, *P* = 0.001 and Fig. 4Bc, *P* = 0.001).

**Fig. 5:**
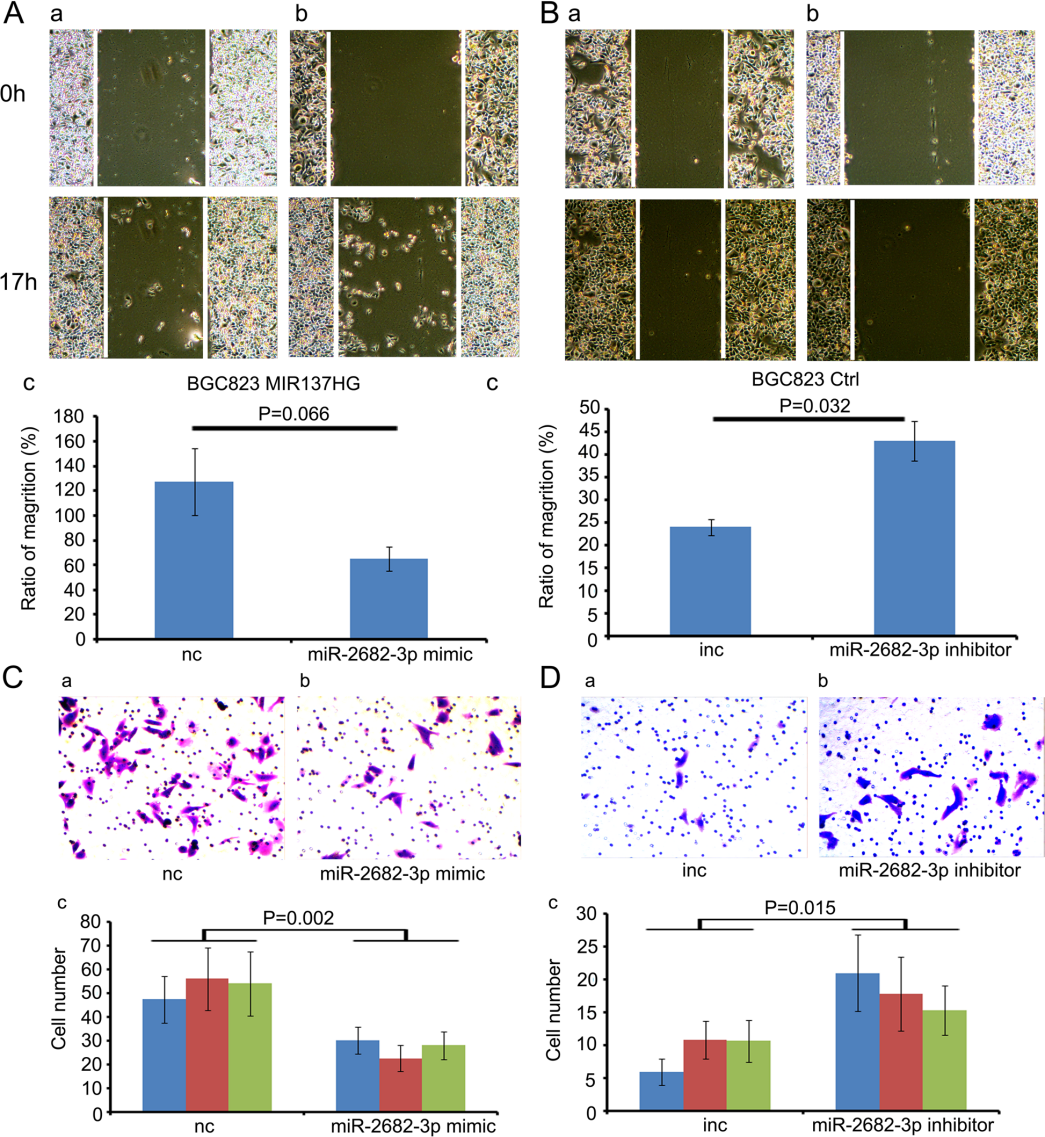
The function of miR-2682-3p and MIR137HG on the migration ability of BGC823. **A** and **B** The scratch assay indicated that miR-2682-3p mimic could inhibit the migration ability of BGC823 MIR137HG (*P* = 0.066), while miR-2682-3p inhibitor could promote the migration ability BGC823 Ctrl (*P* = 0.032). **C** and **D** The transwell assay indicated miR-2682-3p mimic could inhibit the migration ability of BGC823 MIR137HG (*P* = 0.002), while miR-2682-3p inhibitor could promote the migration ability of BGC823 Ctrl (*P* = 0.015)

Because miR-2682-3p expressed in BGC823 MIR137HG was relatively lower than in BGC823 Ctrl, we selected BGC823 MIR137HG cell strain to transfect miR-2682-3p mimic to observe the functional relationships between miR-2682-3p and MIR137HG. The colony formation data showed that miR-2682-3p mimic could inhibit the proliferation of BGC823 MIR137HG overexpression cell strain (Fig. 4C(a,b,c)). Furthermore, the scratch assay and the transwell assay showed that miR-2682-3p mimic could inhibit the ability of migration (Fig. 5A(a,b,c) and Fig. 5C(a,b,c)). On the contrary, we selected miR-2682-3p inhibitor to transfect into BGC823 Ctrl (miR-2682-3p expressed relatively higher than in BGC823 MIR137HG). The colony formation showed that miR-2682-3p inhibitor could promote the proliferation of BGC823 MIR137HG overexpression cell strain (Fig. 4D(a,b,c)). In addition, the scratch assay and the transwell assay showed that miR-2682-3p inhibitor could promote the ability of migration (Fig. 5B(a,b,c) and D(a,b,c)).

**Fig. 6:**
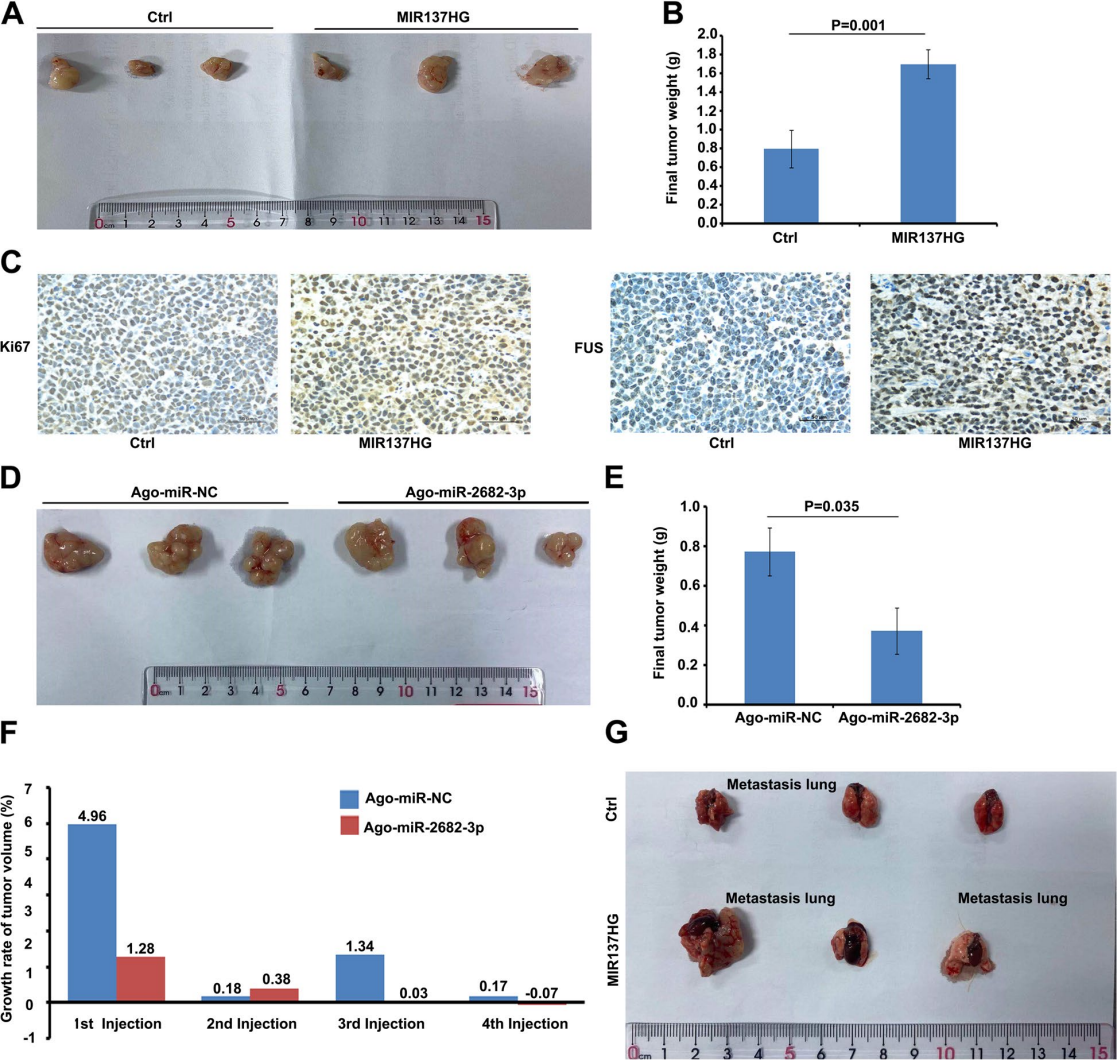
Animal models in nude mice. **A** Photographs of xenograft tumors after sacrifice: Ctrl VS MIR137HG; **B** Weight of xenograft tumors (*n* = 3, *P* = 0.001); **C** Representative photographs of immunohistochemical staining of KI67 and FUS in xenograft tumors. (Scale bar = 50 μm). **D** Photographs of xenograft tumors formed by intratumoral injection after sacrifice (Ago-miR-NC VS Ago-miR-2682-3p). **E** Final volume weight of xenograft tumors formed by intratumoral injection (*n* = 3, *P* = 0.035); **F** Comparing the growth rate of tumor volume treated by Ago-miR-NC and Ago-miR-2682-3p. **G** Photograph of lung metastasis tumors after sacrifice (MIR137HG VS Ctrl, 2/3 VS 1/3)

We separately hypodermic injected Ctrl and MIR137HG cell strain into Balb/C Nude mice. 15 days later, we found that the MIR137HG over-expression group could promote tumor growth compared with the Ctrl group (Fig. 6A and B, Weight, Ctrl vs MIR137HG, *P* = 0.001). IHC data showed that Ki67 and FUS were expressed in the MIR137HG cell strain higher than in the Ctrl cell strain (Fig. 6C). To analyze whether miR-2682-3p could insist the function of MIR137HG, we further injected MIR137HG over-expression BGC823 cell strain into Balb/C nude mice to form the subcutaneously implanted tumor model. We then injected the Ago-miR-2682-3p into the tumor every two days. The results showed that miR-2682-3p could significantly disturb the tumor formation compared with the NC group (Fig. 6D and E, Weight, Ago-miR-NC vs Ago-miR-2682-3p, *P* = 0.035). In addition, the growth rates of tumor volume after injection were slowed down by miR-2682-3p (Fig. 6F). These results indicated that miR-2682-3p could inhibit the function of MIR137HG in tumor formation. The tail injection of Balb/C nude mice showed that MIR137HG could promote lung metastasis compared with the Ctrl group. (Fig. 6G, lung metastasis: MIR137HG VS Ctrl, 2/3 VS 1/3).

### FUS was the exact target of MIR137HG and miR-2682-3p

**Fig. 7:**
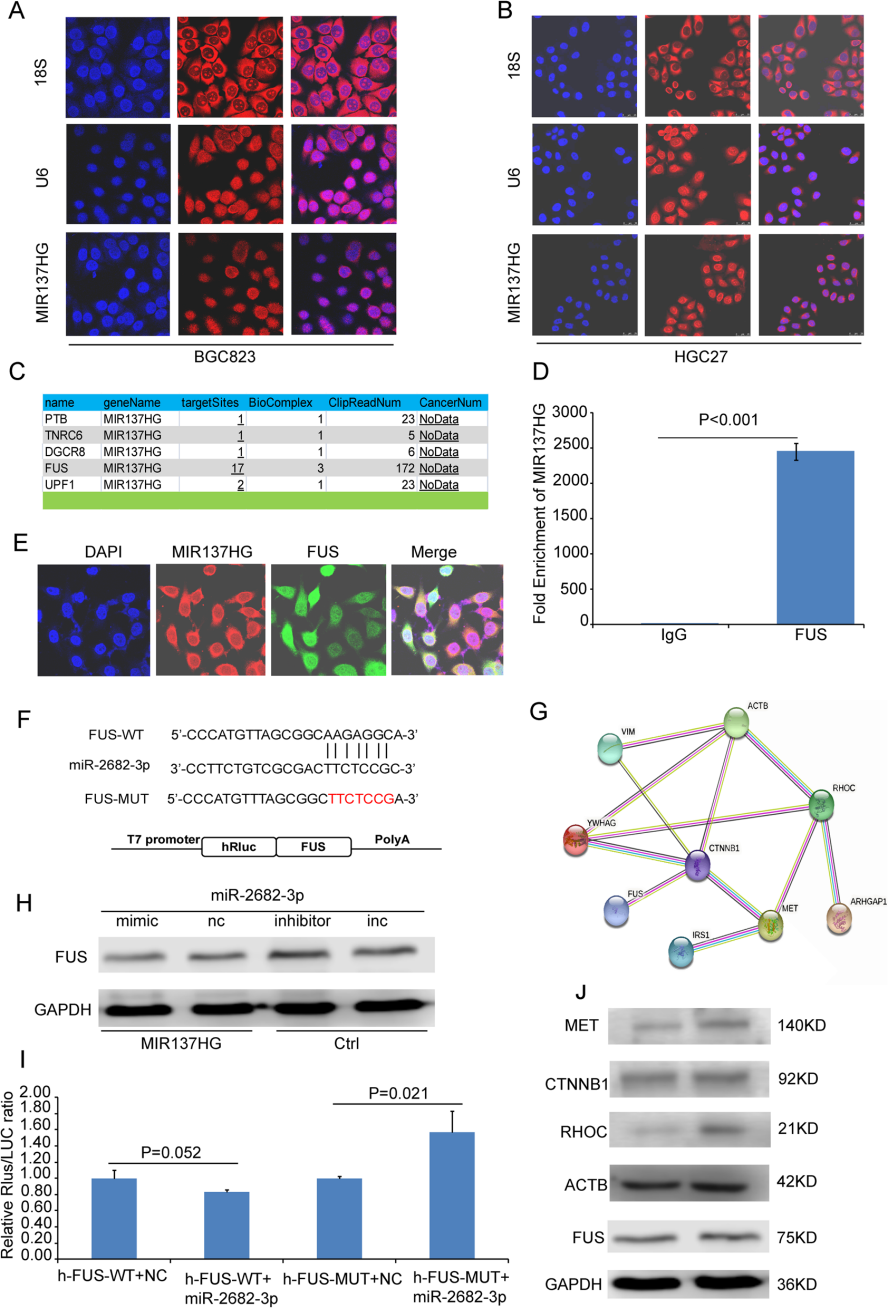
The subcellular location of MIR137HG and the interaction among molecules. **A** The subcellular location of MIR137HG BGC823; **B** The subcellular location of MIR137HG HGC27 ( DAPI was used to stain the nucleus; Cy3 separately labeled MIR137HG, U6, and 18S; U6 was the control of nucleus sub-location; 18S was the control of cytoplasm); **C** Starbase V2.0 indicated that FUS was a candidate target of MIR137HG; **D** RIP assay showed that FUS could directly interact with MIR137HG; **E** Con-focus data showed that MIR137HG and FUS could sub-locate in the same region of the cell; **F** TargetScan predicted that miR-2682-3p could target with FUS; **G **The String database predicted the relationships among FUS and its candidated targets tested by IP followed LC-MS/MS; **H** Western blot data showed that miR-2682-3p mimic could inhibit the expression of FUS in BGC823 MIR137HG, while miR-2682-3p inhibitor could promote the expression of FUS in BGC823 Ctrl; **I** Dual-luciferase assay showed that miR-2683-3p could bind data showed that MET and RHOC could co-express with FUS; **J** Western blot data showed the expression of MET, CTNNB1, RHOC, ACTB (ACTIN labelled in the primary gel picture), FUS in BGC823 Ctrl and BGC823 MIR137HG

To further explore the mechanism of MIR137HG, we separately observed the subcellular location of MIR137HG in BGC823 and HGC27. Data showed that MIR137HG was sub-localized in both the nucleus and cytoplasm (Fig. 7A and B).

The Starbase V2.0 has predicted 5 candidate targets for MIR137HG (Fig. 7C) [23, 24]. RIP assay implied that MIR137HG could directly interact with FUS (Fig. 7D). In addition, co-location performed by MIR137HG RNA-FISH and FUS immunofluorescence showed that MIR137HG and FUS could co-localize in the BGC823 cell line (Fig. 7E).

TargetScan database showed that FUS might be the candidate target of miR-2682-3p (Fig. 7F). Western blot assay indicated that FUS expression could be decreased by miR-2682-3p mimic, while it could be increased by a miR-2682-3p inhibitor (Fig. 7H). The data of the dual-luciferase reporter assay confirmed that FUS interacted with miR-2682-3p (Fig. 7I). We also tested the downstream differentiation of protein expression between BGC823 Ctrl and BGC823 MIR137HG by IP target FUS following LC–MS/MS (Supplement datasets file). And we selected several proteins related to tumor growth and metastasis to analyze further. String data showed that they might have more contact with each other (Fig. 7G). Western blot data showed that MET, CTNNB1 and RHOC were also co-expressed with the over-expression MIR137HG (Fig. 7J, Supplement Fig. 3, Supplement Fig. 4).

## Discussion

Gastric cancer is a severe threat to human health. However, the mechanisms were still complicated and have not been revealed. Recently, ncRNAs have been proven to play a critical role in various cancers. Our study illustrated long non-coding RNA MIR137HG could promote the progression of gastric cancer through interacting with FUS and affecting a series of its downstream molecules (MET, RHOC, and CTNNB1. et al.) which might regulate the cell survival, proliferation, migration, and invasion. On the contrary, its embedded miR-2682-3p could disturb the function of MIR137HG to gastric cancer by holding the same target FUS.

We firstly focused on MIR137HG because of miR-137, which we tested and reported as a tumor suppresser molecule in gastric cancer [25, 26]. As miR-137 is located in the third exon of MIR137HG, we firstly supposed that MIR137HG simply played as the primary molecule of miR-137. However, the results showed that the expression of MIR137HG was higher in GC samples than in SM samples and was not related to the expression of miR-137 in gastric cancer (data not shown). Genecard database indicated that MIR137HG might have 6 kinds of transcripts, which have been numbered as 201–206. It meant that MIR137HG was not the only form of pri-miR-137. That would lead to the non-correlative between their expression level in the tissue. However, the expression coincidence between MIR137HG and miR-137 could be tested in the gastric cell line. That might be related to the gene expression in the cell line. Furthermore, we found another embedded miR-2682 in the second intron of MIR137HG. And the expression of miR-2682-3p was negatively related to MIR137HG, which aroused our interest in their functions and mechanisms.

According to the clinical analysis, MIR137HG was more expressed in male patients than female patients. If it played an oncogene role in GC, it might be one of the reasons for a relatively higher occurrence of GC in males than in females—about 2 ~ 2.5 times in males than in females [4]. More interestingly, MIR137HG was more expressed in the poor differentiation group than in SM’s middle/well differentiation group. We could get some clues from this data: 1. patients with higher MIR137HG expression intending to lead to the poor differentiation tumor. We found a cut-off value that could discriminate the GC patients with poor differentiation from those with moderate/well differentiation. Although it didn’t have a high diagnostic value limit by the present research condition, it meant that we could use the expression of MIR137HG in SM samples from GC patients to predict the malignancy of GC, which may make sense to the therapy of different GC patients. 2. MIR137HG might influence the survival prognosis of GC patients. Unfortunately, we didn’t further track the survival data and could not give a definite answer, which we should avoid and overcome subsequent studies. 3. The significant difference among differentiation in SM indicated that the tumor microenvironment might be related to the function of MIR137HG. The significance of MIR137HG in SM samples of GC patients shift our focus from the tumor itself to the tumor microenvironment. Therefore, we predict that the function of MIR137HG may be involved in the tumor microenvironment. However, the hypothesis still needs to be confirmed by future assays.

According to the results in vivo, MIR137HG could promote gastric cancer cell colony formation, migration, and invasion (Supplement Fig. 1) in the gastric cancer cell lines. Meanwhile, animal models supported the results of cell line assays. MIR137HG can promote tumor formation and lung metastasis. The function results in vivo and in vitro were coincident with expression data, which indicated that MIR137HG played an oncogene in gastric cancer. Some reports indicated that MIR137HG might be related to cancer. For example, Wang et al. reported that the MIR137HG gene polymorphisms were implicated in liver cancer susceptibility among the Chinese Li population [27]. However, few reports were related to the function of MIR137HG in human tumors, which was the significance of our present research.

The subcellular location of molecules determines their functions and mechanisms in cellular. RNA-FISH assay showed that MIR137HG is located both in the nucleus and cytoplasm. That means that it could interact with molecules in the nucleus and cytoplasm. Biological information analysis indicated that MIR137HG could interact with DGCR8 and FUS. DGCR8 is the critical molecular of miRNA maturation [28]. Therefore, it was reasonable that DGCR8 could interact with MIR137HG and be involved in the maturation process of miR-2682 and miR-137. And RIP assay confirmed this prediction (Supplement Fig. 2). FUS is an essential RNA binding protein, which has been reported in nervous system diseases and human tumors. As mentioned earlier, Fent et al. have reported that FUS can activate XIAP and promote the proliferation and migration of prostate cancer by interacting with circ005267 [19]. Therefore, it indicated that FUS might play as an oncogene in human tumors. Our data showed that FUS and MIR137HG could distribute in the same area of cellular, which indicated their possible interaction. And RIP data have confirmed their direct interaction with each other.

Suppose the oncogene role of MIR137HG and its interactive protein FUS, and we tried to explore their downstream molecules. We performed an IP assay and compared the protein expression differences between BGC823 Ctrl and BGC823 MIR137HG. LC/MS provided about 116 proteins with significant differences between them. Among them, we selected several proteins related to the progression of cancer. And data of Western blot have confirmed that RHOC, MET, CTNNB1, and FUS expressed coincident with MIR137HG. According to the current acknowledgment, MET might regulate GC cell survival and proliferation by the Wnt signaling pathway. In contrast, RHOC might regulate the GC cell metastasis by the mTOR signaling pathway. However, a series of assays should be verified in the following assays.

According to the negative relationship between miR-2682-3p and MIR137HG, they might have opposite functions. Data showed that miR-2682-3p played a tumor suppressor function in GC cancer in vivo and in vitro, which confirmed the negative relationship between miR-2682-3p and MIR137HG. Some reports were also consistent with our conclusion. For example, miR-2682-3p has been reported as a tumor suppressor gene in osteosarcoma [15]. Moreover, FUS could not only interact with MIR137HG but also was predicted by TargetScan as a candidate target of miR-2682-3p. Data of western blot assay showed that miR-2682-3p mimic could decrease the protein level of FUS, while miR-2682-3p inhibitor could increase the protein level of FUS. And Dual-luciferase reporter assay has confirmed such a prediction.

## Conclusions

**Fig. 8:**
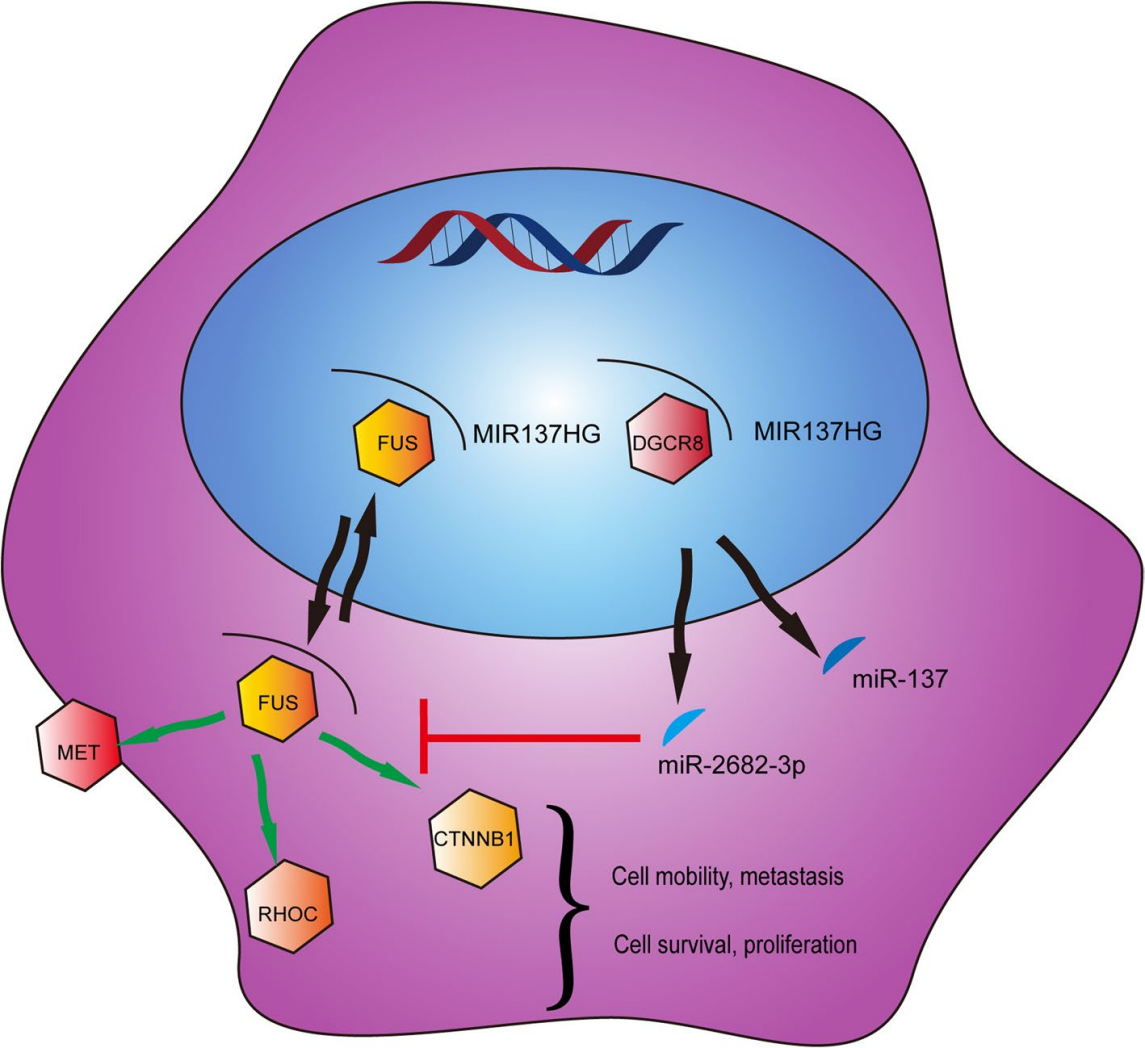
Structure chart of the role of the MIR137HG/FUS/miR-2682-3p axis in gastric cancer progression. Our combined data indicated a model whereby MIR137HG promotes the advancement of gastric cancer through interacting with FUS, followed by influencing a series downstream related proteins (MET, RHOC, CTNNB1) and finally affecting GC progression. miR-2682-3p could inhibit the function of MIR137HG by targeting FUS

In summary, our data indicated the following conclusions: 1) MIR137HG can be spliced by DGCR8 and then produced miR-137 and miR-2682; 2) FUS can interact with MIR137HG and can shuttle back and forth between nucleus and cytoplasm to influence a series of proteins (MET, RHOC, CTNNB1. et al.) and finally promote GC progression; 3) miR-2682-3p can play as an antagonistic role to inhibit the progression by inhibiting the exact target of MIR137HG, FUS (Fig. 8).

## Supplementary Information

**Additional file 1: Supplement Figure 1.** The invasion assay in BGC823 cell line. A BGC823 Ctrl; B. BGC823 MIR137HG; C. Comparation of the invasion cell number between BGC823 Ctrl and BGC823 MIR137HG.

**Additional file 2: Supplement Figure 2.** RIP assay showed that DGCR8 could directly interact with MIR137HG.

**Additional file 3: Supplement Figure 3.** The gel initial picture obtained by protein imaging system for Figure 7H and Figure 7J. The expression of FUS and GAPDH in BGC823 Ctrl and BGC823 MIR137HG cells were cropped from this picture and combined with other gel pictures of Figure 7J.

**Additional file 4: Supplement Figure 4.** The gel initial picture obtained by protein imaging system for Figure 7J. The expression of MET, CTNNB1, RHOC and ACTB (labelled as ACTIN in original gel picture) in BGC823 Ctrl and BGC823 MIR137HG cells were cropped from this picture.

**Additional file 5.** Supplement datasets file. Datasets about candidated proteins might be the downstream targets which could direct or indirect interacted with FUS by IP following with gel strip related LC-MS/MS.

## Data Availability

1. Supplement information file which contained the Supplement Figs. 1, 2, 3, 4. 2. Supplement datasets file which only contain the results section of LC-MS/MS. And the whole data of LC-MS/MS has been uploaded onto the PRIDE, which will be accessible after publication of the manuscript. Currently, part of the data is present under result section in the Supplement datasets file uploaded as an excel file. 3. The mass spectrometry proteomics data (Supplement datasets file) have been deposited to the ProteomeXchange Consortium via the PRIDE partner repository with the dataset identifier PXD031258 and 10.6019/PXD03125.

## Abbreviations

GC: Gastric Cancer
SM: Surgical Margin
FUS: Fused in Sarcoma/Translocated in Sarcoma
lncRNA: Long non-coding RNA
miRNA: MicroRNA
RHOC: Ras homolog gene family, member C
MET: MET-proto-oncogene, receptor tyrosine kinase
CTNNB1: Catenin, beta1
