# Correction: miR-2682-3p antagonizes its host lncRNA-MIR137HG by interacting with the same target FUS to regulate the progression of gastric cancer

**DOI:** 10.1186/s12885-022-10365-1

**Published:** 2022-11-29

**Authors:** Yantao Du, Yichen Chen, Tao Wu, Xiaodan Fan, Wei Lin, Zhouhua Jiang

**Affiliations:** 1 The Afliated Hospital of Medical School of Ningbo University, Renmin Road No.247, Ningbo, 315020 Zhejiang China; 2 Ningbo Institute of Medical Science, Yangshan Road No.42-46, Ningbo, 315020 Zhejiang China; 3 Medical School of Ningbo University, Fenghua Road No.818, Ningbo, 315211 Zhejiang China; 4 Zhejiang Pharmaceutical College, Ningbo, 315100 ZhejiangZhejiang China; 5 Ningbo Medical Centre Lihui Li Eastern Hospital, Ningbo University, Jiangnan Road No.1111, Ningbo, 330212 Zhejiang China; 6 Ningbo Women and Children Hospital, Ningbo Liuting Road No.339, Ningbo, 315012 Zhejiang China

**Correction: BMC Cancer 22, 689 (2022)**

**https://doi.org/10.1186/s12885-022-09740-9**

Following publication of the original article [1], the authors reported the following errors:

1. The results of colony formation assay which have been used in Fig. 4C(c) and Fig. 4D(c) were accidentally used again in Fig. 5A(c) and Fig. 5B(c). The authors corrected Fig. 5A(c) and Fig. 5B(c) in this correction article.
2. The western blot result annotations "Ctrl "and "MIR137HG" were missed in Fig. 7J during the editing process. The authors added the missed annotations in this correction.

The correct Fig. 5 and Fig. 7 are given below:

**Fig. 5:**
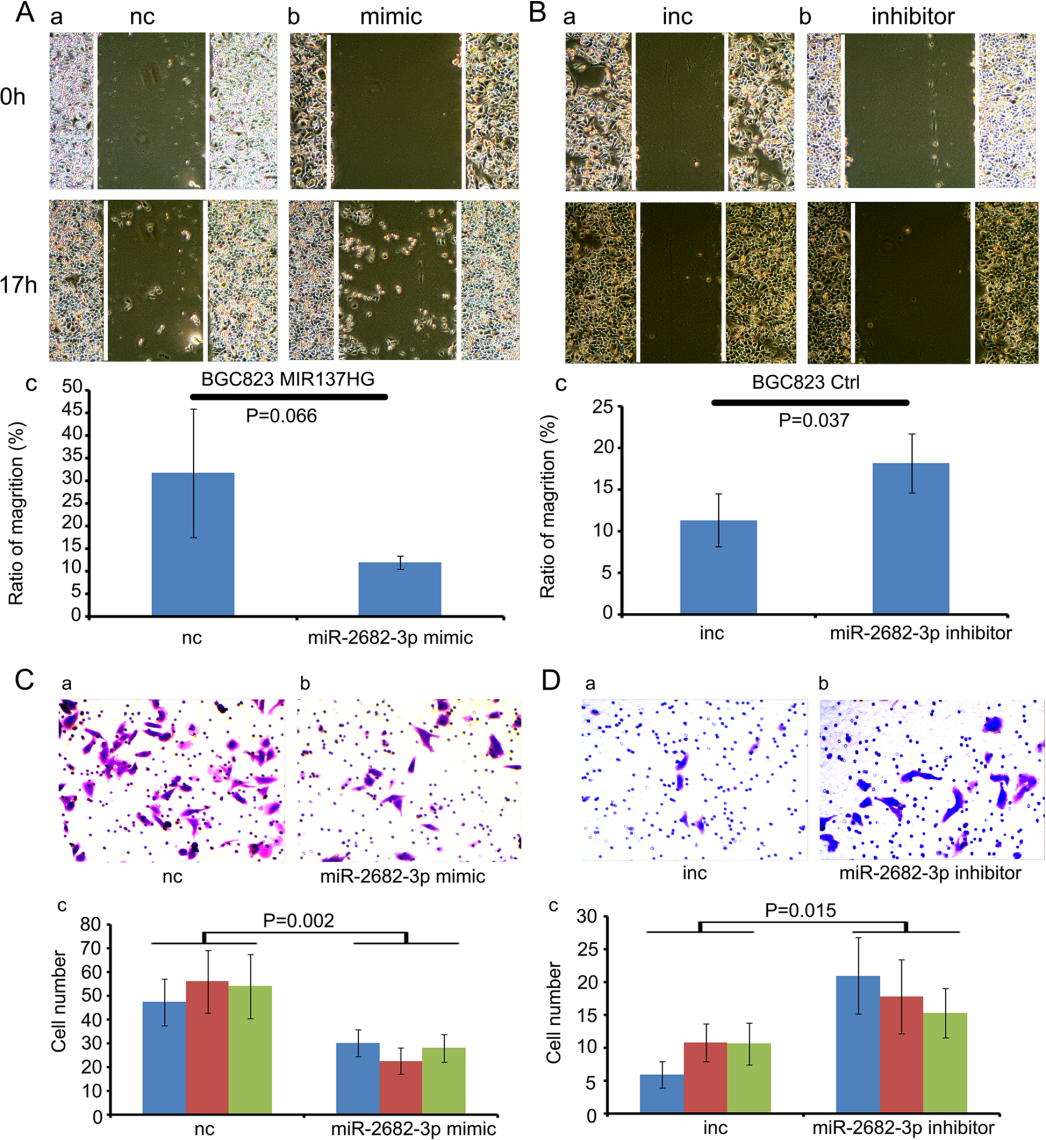
The function of miR-2682-3p and MIR137HG on the migration ability of BGC823. **A** and **B** The scratch assay indicated that miR-2682-3p mimic could inhibit the migration ability of BGC823 MIR137HG (*P* = 0.066), while miR-2682-3p inhibitor could promote the migration ability BGC823 Ctrl (*P* = 0.037). **C** and **D** The transwell assay indicated miR-2682-3p mimic could inhibit the migration ability of BGC823 MIR137HG (*P* = 0.002), while miR-2682-3p inhibitor could promote the migration ability of BGC823 Ctrl (*P* = 0.015)

**Fig. 7:**
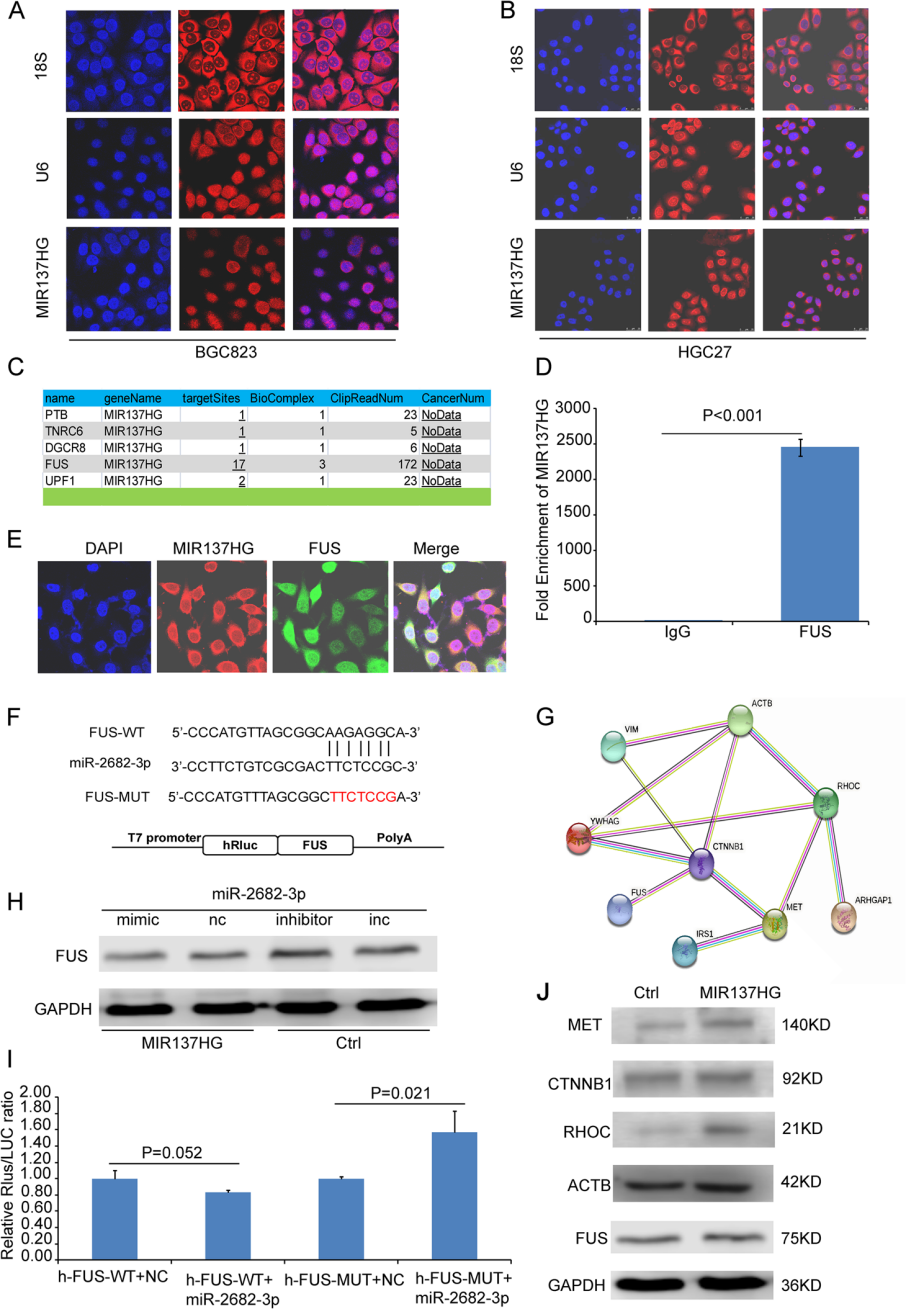
The subcellular location of MIR137HG and the interaction among molecules. **A** The subcellular location of MIR137HG BGC823; **B** The subcellular location of MIR137HG HGC27 ( DAPI was used to stain the nucleus; Cy3 separately labeled MIR137HG, U6, and 18S; U6 was the control of nucleus sub-location; 18S was the control of cytoplasm); **C** Starbase V2.0 indicated that FUS was a candidate target of MIR137HG; **D** RIP assay showed that FUS could directly interact with MIR137HG; **E** Con-focus data showed that MIR137HG and FUS could sub-locate in the same region of the cell; **F** TargetScan predicted that miR-2682-3p could target with FUS; **G** The String database predicted the relationships among FUS and its candidated targets tested by IP followed LC-MS/MS; **H** Western blot data showed that miR-2682-3p mimic could inhibit the expression of FUS in BGC823 MIR137HG, while miR-2682-3p inhibitor could promote the expression of FUS in BGC823 Ctrl; **I** Dual-luciferase assay showed that miR-2683-3p could bind data showed that MET and RHOC could co-express with FUS; **J** Western blot data showed the expression of MET, CTNNB1, RHOC, ACTB (ACTIN labelled in the primary gel picture), FUS in BGC823 Ctrl and BGC823 MIR137HG
