# Supplementary figures and images for: miR-2682-3p antagonizes its host lncRNA-MIR137HG by interacting with the same target FUS to regulate the progression of gastric cancer
Source: BMC Cancer. 2022 Jun 22;22:689. doi: 10.1186/s12885-022-09740-9 (PMC9219209; doi:10.1186/s12885-022-09740-9)

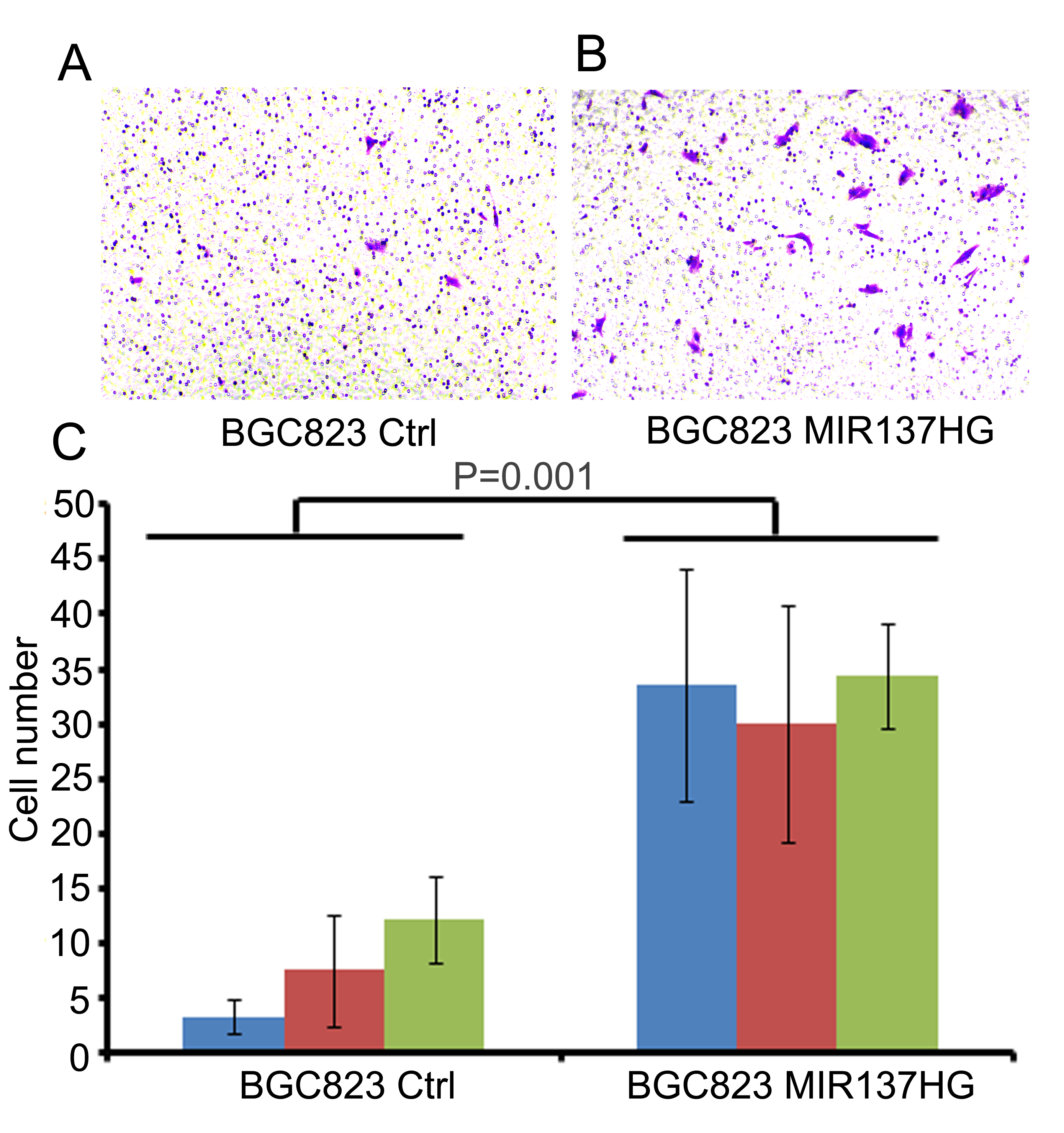

Supplement: Supplementary file 1 — Additional file 1: Supplement Figure 1. The invasion assay in BGC823 cell line. A BGC823 Ctrl; B. BGC823 MIR137HG; C. Comparation of the invasion cell number between BGC823 Ctrl and BGC823 MIR137HG. [file 12885_2022_9740_MOESM1_ESM.tif]

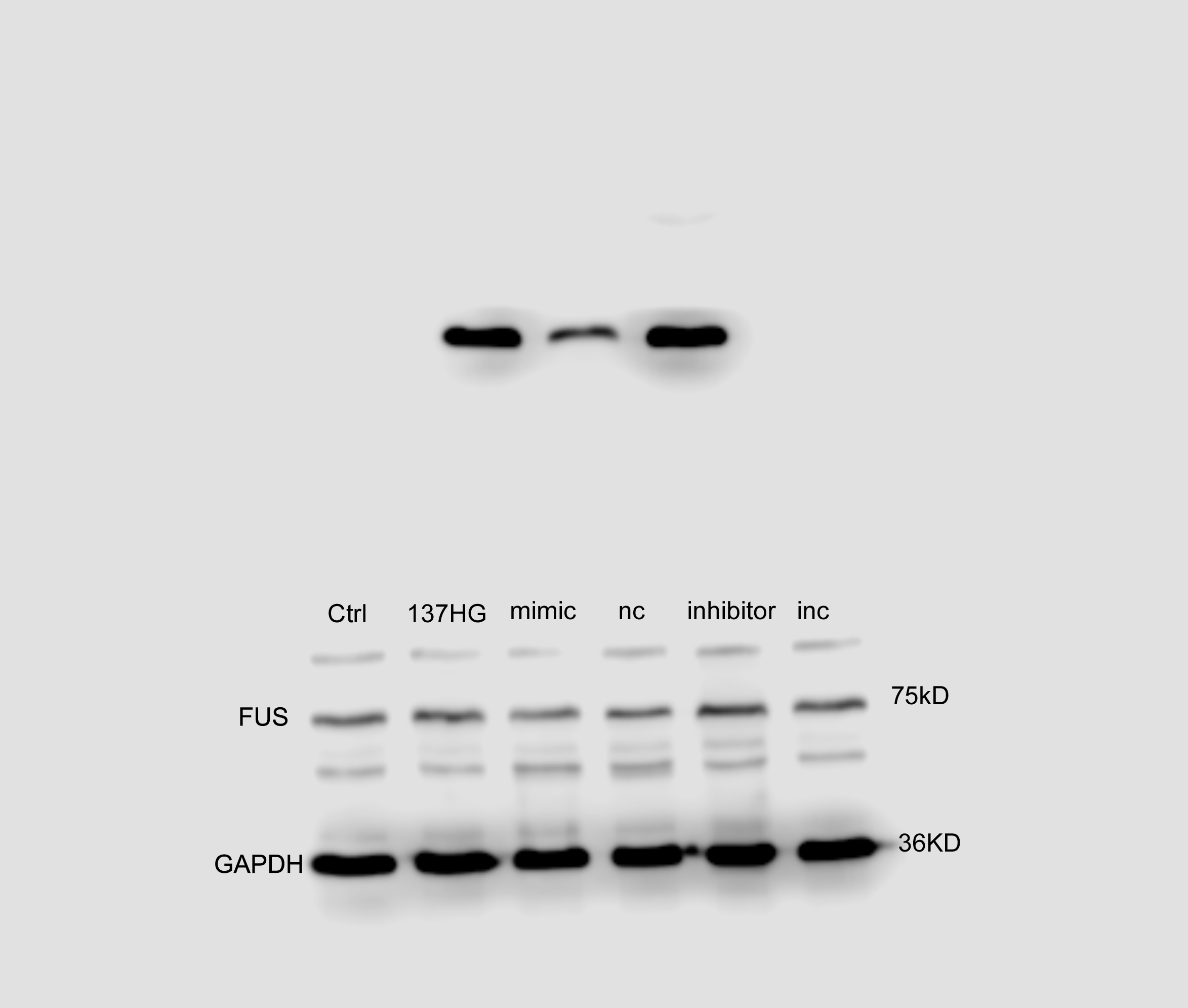

Supplement: Supplementary file 3 — Additional file 3: Supplement Figure 3. The gel initial picture obtained by protein imaging system for Figure 7H and Figure 7J. The expression of FUS and GAPDH in BGC823 Ctrl and BGC823 MIR137HG cells were cropped from this picture and combined with other gel pictures of Figure 7J. [file 12885_2022_9740_MOESM3_ESM.tif]

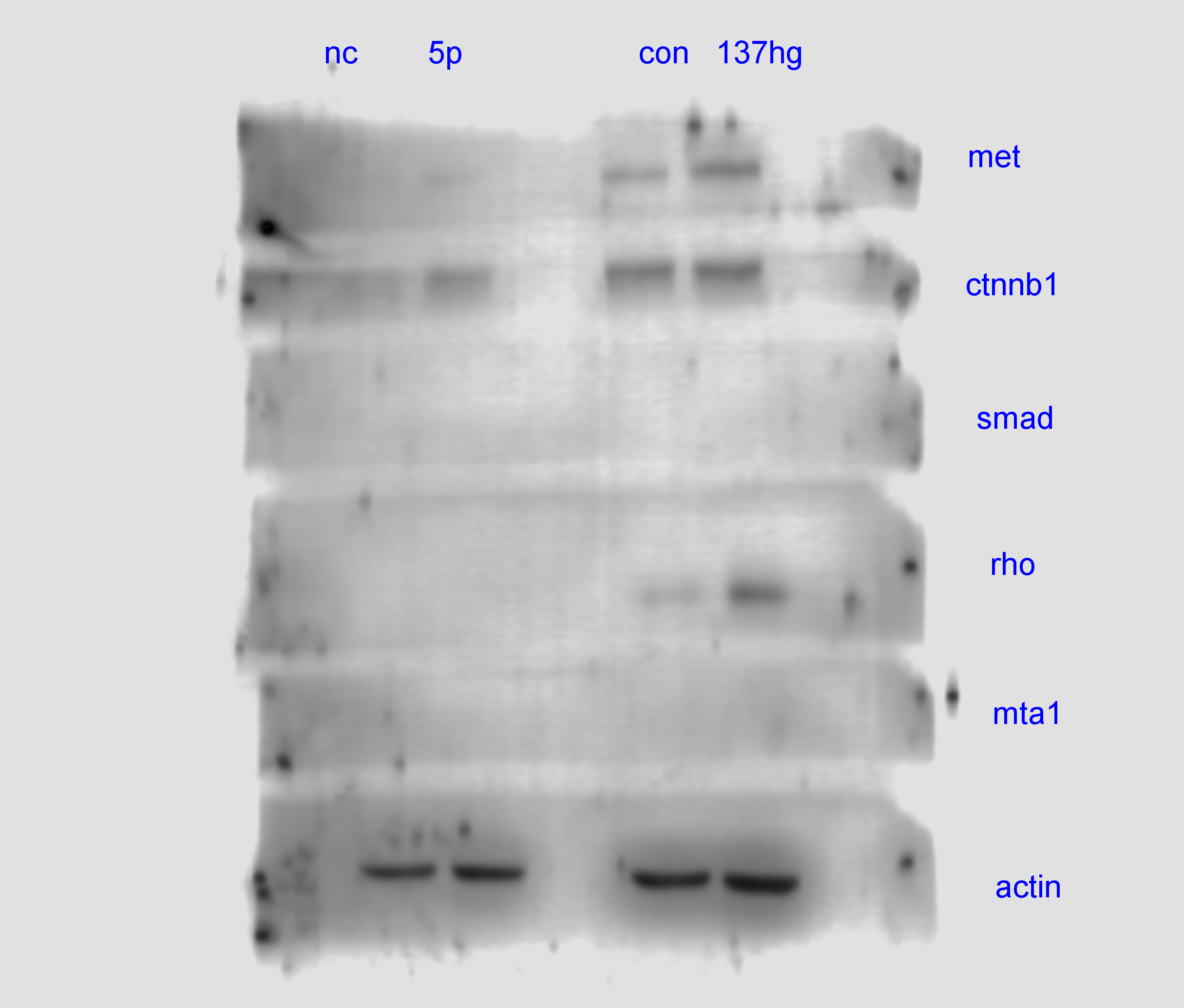

Supplement: Supplementary file 4 — Additional file 4: Supplement Figure 4. The gel initial picture obtained by protein imaging system for Figure 7J. The expression of MET, CTNNB1, RHOC and ACTB (labelled as ACTIN in original gel picture) in BGC823 Ctrl and BGC823 MIR137HG cells were cropped from this picture. [file 12885_2022_9740_MOESM4_ESM.tif]
